# Supplementary material for: Risk factor of elevated matrix metalloproteinase-3 gene expression in synovial fluid in knee osteoarthritis women
Source: PLoS One. 2023 Mar 31;18(3):e0283831. doi: 10.1371/journal.pone.0283831 (PMC10065264; doi:10.1371/journal.pone.0283831)
Supplement: S1 Data — (PDF) [file pone.0283831.s001.pdf]

Row Research Data

Risk Factor of Elevated Matrix Metalloproteinase-3 Gene Expression in Synovial Fluid In Knee Osteoarthritis Women

No	Patient code	Age	Education	Occupational Status	Weight	Height	BMI	Nutritional status	OA degree	Menopause Status	IL1 Level	TNF Level	Gen MMP3 rs679620	Polimorfism	Gene expression
1	1b	59	diploma	civil servant	150	62.7	27.9	obesity	2	(-)	356.339	95.083	AG	Mutan	1.29
2	2b	57	Senior high school	Housewife	155	69.2	28.8	obesity	3	(-)	204.027	64.273	AG	Mutan	1.6
3	3b	53	diploma	Housewife	152	47.1	20.4	Normal	2	(-)	248.737	61.606	AG	Mutan	1.02
4	5b	64	elementary school	Housewife	150	71	31.6	obesity	2	(-)	1084.889	215.826	GG	Mutan	2.33
5	6b	61	Senior high school	Retired	150	66.3	29.5	obesity	2	(-)	267.404	114.156	AG	Mutan	17.29
6	7	70	Senior high school	Housewife	157	65.8	26.8	obesity	3	(-)	573.856	142.721	GG	Mutan	13.12
7	8b	54	Senior high school	Housewife	145	53	25.2	obesity	3	(-)	420.142	126.025	AA	Wild Type	1.22
8	9b	40	diploma	Housewife	154	66	27.9	obesity	2	(+)	487.928	147.602	GG	Mutan	3.22
9	10b	64	Junior high school	Housewife	145	55.4	26.4	obesity	3	(-)	284.883	58.139	AG	Mutan	0.68
10	11b	71	Senior high school	Housewife	159	55	21.7	Normal	3	(-)	316.569	68.931	GG	Mutan	1.54
11	12b	64	Junior high school	Housewife	152	58.3	25.2	obesity	3	(-)	438.806	84.982	GG	Mutan	2.41
12	13b	55	Senior high school	civil servant	154	58	24.5	Overweight	2	(-)	297.767	72.911	AG	Mutan	6.07
13	4b	51	elementary school	Housewife	165	72	26.5	obesity	3	(-)	360.818	72.207	GG	Mutan	0.61
14	15b	59	elementary school	Housewife	155	68	28.3	obesity	4	(-)	500.818	59.409	AA	Wild Type	1.09
15	14b	63	elementary school	Housewife	152	67	29	obesity	3	(-)	297.767	85.459	GG	Mutan	7.44
16	17b	59	Junior high school	Housewife	150	72	32	obesity	2	(-)	372.319	80.105	AA	Wild Type	1.67
17	18b	62	Senior high school	Housewife	144	62.7	30.3	obesity	2	(-)	448.212	98.12	AA	Wild Type	1.82
18	20b	67	Senior high school	Housewife	150	66.7	29.6	obesity	2	(-)	550.495	79.513	AG	Mutan	10.12
19	21b	73	elementary school	Housewife	145	48	22.9	Normal	2	(-)	330.696	81.886	AG	Mutan	0.67
20	22b	62	bachelor	Retired	151	63	27.6	obesity	2	(-)	535.622	104.239	AG	Mutan	4.83
21	23b	61	Junior high school	Housewife	142	51	25.3	obesity	3	(-)	404.225	95.568	GG	Mutan	3.06
22	24b	44	diploma	civil servant	165	85	30.9	obesity	3	(+)	1247.062	309.092	GG	Mutan	1.27
23	25b	76	Junior high school	Housewife	139.5	52	26.7	obesity	4	(-)	439.328	93.873	AG	Mutan	44.97
24	27b	69	elementary school	Housewife	154.5	80.7	33.8	obesity	3	(-)	616.261	125.642	AG	Mutan	19.62

25	28b	63	bachelor	Retired	147	51	23.2	Overweight	3	(-)	673.612	145.882	GG	Mutan	1.76
26	29b	56	elementary school	Housewife	156	65	27.1	obesity	3	(-)	769.697	187.441	GG	Mutan	0.4
27	30b	54	Senior high school	Housewife	170	71.6	24.8	Overweight	3	(+)	208.072	50.21	GG	Mutan	6.05
28	31b	53	Senior high school	Housewife	159	74	29.3	obesity	4	(-)	355.842	62.881	GG	Mutan	7.98
29	32b	60	elementary school	Housewife	160	64	25	obesity	4	(-)	169.501	34.252	AG	Mutan	38.7
30	33b	67	Junior high school	Housewife	150	55	24.4	Overweight	3	(-)	204.925	61.49	GG	Mutan	1.1
31	34b	76	elementary school	Housewife	157	55	22.4	Normal	3	(-)	405.247	78.448	AG	Mutan	38.7
32	35b	63	Junior high school	Housewife	148.5	53.7	24.5	Overweight	4	(-)	309.311	55.26	AG	Mutan	108.33
33	36b	51	bachelor	Housewife	160	80	31.3	obesity	3	(+)	259.908	57.793	GG	Mutan	2.12
34	37b	60	bachelor	Retired	156	69	28.8	obesity	3	(-)	965.75	151.993	GG	Mutan	16.16
35	38b	54	bachelor	civil servant	156.5	70.8	28.9	obesity	2	(-)	204.925	31.069	AG	Mutan	12.11
36	39b	56	elementary school	trader	156	65	26.8	obesity	3	(-)	211.226	43.584	GG	Mutan	3.09
37	1	71	bachelor	civil servant (gu	159	55	21.8	Normal	2	(-)	443.503	99.461	AG	Mutan	0.61
38	2	77	elementary school	Housewife	159	65	25.7	obesity	3	(-)	585.64	143.115	AA	Wild Type	0.18
39	3	40	elementary school	Housewife	160	77	30.1	obesity	2	(+)	245.033	82.123	GG	Mutan	4.42
40	4	68	elementary school	Housewife	146	50	23.5	Overweight	3	(-)	267.404	76.441	AG	Mutan	1.38
41	6	47	diploma	Housewife	157	65	26.4	obesity	2	(+)	463.468	104.608	AG	Mutan	0.77
42	9	59	Senior high school	Housewife	155	79	32.9	obesity	2	(-)	317.054	75.969	AG	Mutan	0.25
43	10	56	Junior high school	Housewife	155	55	22.9	Normal	2	(-)	403.203	105.593	AG	Mutan	41.41
44	11	56	Junior high school	trader	151	69	30.3	obesity	2	(-)	609.417	146.676	GG	Mutan	14.12
45	12	61	elementary school	Housewife	145	45	21.4	Normal	2	(-)	530.69	113.781	AA	Wild Type	36.71
46	13	54	elementary school	Housewife	155	76	31.7	obesity	3	(+)	368.31	62.533	AA	Wild Type	24.46
47	14	52	master	civil servant (gu	151	59	25.9	obesity	2	(-)	313.662	58.716	GG	Mutan	0.37
48	15	63	Junior high school	Housewife	150	56	24.9	Overweight	3	(-)	172.123	67.414	AG	Mutan	57.06
49	16	56	Senior high school	Housewife	162	75	28.6	obesity	2	(-)	489.534	138.533	GG	Mutan	0.16
50	17	69	diploma	civil servant (gu	150	59	26.2	obesity	3	(-)	307.863	54.34	GG	Mutan	6.85
51	19	41	bachelor	civil servant (gu	160	80	31.3	obesity	3	(+)	337.069	84.982	AG	Mutan	26.72
52	20	61	Senior high school	Housewife	150	85	37.8	obesity	3	(-)	458.193	82.718	GG	Mutan	3.03
53	21	68	Junior high school	Housewife	155	53	22.1	Normal	2	(-)	332.654	79.276	GG	Mutan	12.19

54	22	75	Senior high school	Housewife	170	78	26.9	obesity	3	(-)	344.454	57.909	GG	Mutan	26.18
55	23	54	diploma	Housewife	162	67	25.6	obesity	2	(-)	475.658	111.288	AA	Wild Type	0.67
56	24	80	Senior high school	Housewife	155	55	22.9	Normal	2	(-)	254.312	67.647	GG	Mutan	32.92
57	25	60	Senior high school	Housewife	155	58	24.2	Overweight	2	(-)	381.375	122.337	GG	Mutan	5.48
58	26	48	elementary school	Housewife	159	84	33.2	obesity	2	(+)	594.1	152.661	AG	Mutan	11.16
59	27	44	elementary school	Housewife	155	74	30.8	obesity	2	(+)	416.02	106.086	GG	Mutan	1.72
60	28	56	bachelor	Housewife	158	54	21.7	Normal	2	(-)	463.996	155.745	GG	Mutan	20.51
61	29	48	master	civil servant	155	59	24.6	Overweight	2	(+)	308.828	101.294	AG	Mutan	0.87
62	30	66	elementary school	Housewife	160	72	28.1	obesity	3	(-)	636.357	222.478	AG	Mutan	19.68
63	31	70	diploma	Housewife	150	62	27.6	obesity	3	(-)	295.373	70.684	AG	Mutan	9.41
64	32	70	bachelor	Retired	150	55	24.4	Overweight	3	(-)	624.276	198.383	AG	Mutan	17.86
65	34	47	Senior high school	Housewife	158	55	22.1	Normal	3	(+)	140.199	60.334	GG	Mutan	2.85
66	35	55	elementary school	Housewife	152	62	26.9	obesity	2	(-)	434.122	143.51	GG	Mutan	5.76
67	37	65	elementary school	Housewife	160	66	25.8	obesity	2	(-)	318.996	96.782	AG	Mutan	2.22
68	38	52	Senior high school	Housewife	156	70	29.2	obesity	2	(-)	392.508	105.962	AG	Mutan	3.83
69	39	54	bachelor	Housewife	145	99	47.1	obesity	2	(-)	607.14	214.776	AG	Mutan	2.01
70	40	61	Senior high school	Housewife	150	75	33.3	obesity	2	(-)	424.79	130.249	AG	Mutan	46.44
71	41	54	bachelor	civil servant	156	80	32.9	obesity	2	(+)	421.69	130.378	AG	Mutan	30.23
72	42	72	Junior high school	Housewife	150	50	22.2	Normal	3	(-)	752.574	229.676	AG	Mutan	13.95
73	45	68	Senior high school	Retired	156	74	30.8	obesity	3	(-)	339.527	82.837	AG	Mutan	45.5
74	48	55	Senior high school	Housewife	147	64	29.6	obesity	3	(-)	449.784	82.837	AG	Mutan	5.09
75	49	67	bachelor	Retired	150	57	25.3	obesity	3	(-)	715.126	142.458	AA	Wild Type	1.81
76	50	74	Senior high school	Housewife	162	67	25.8	obesity	2	(-)	456.613	129.864	GG	Mutan	7.28
77	51	57	elementary school	Housewife	169	72	25.2	obesity	2	(-)	375.835	96.418	AG	Mutan	24.39
78	53	48	bachelor	civil servant	151	75	32.9	obesity	2	(-)	455.561	168.396	GG	Mutan	63.99
79	54	81	elementary school	Housewife	155	53.6	22.3	Normal	2	(-)	377.344	106.703	GG	Mutan	0.93
80	55	63	Senior high school	Housewife	155	63.8	26.6	obesity	2	(-)	321.913	103.133	AG	Mutan	86.89
81	56	87	Senior high school	Retired	150	62.5	27.8	obesity	3	(-)	297.767	114.781	AG	Mutan	14.49
82	57	60	Senior high school	Housewife	151	63.6	27.9	obesity	2	(-)	557.696	157.226	AG	Mutan	47.97

83	58	59	bachelor	Retired	156	69.3	28.5	obesity	2	(-)	374.327	118.799	GG	Mutan	1.82
84	59	78	Junior high school	Retired	160	55	21	Normal	2	(-)	745.275	148	GG	Mutan	39.28
85	60	56	elementary school	Housewife	153	63	26.9	obesity	2	(-)	718.722	204.38	GG	Mutan	47.27
86	62	79	elementary school	Housewife	149	57	25.9	obesity	2	(-)	273.05	60.565	GG	Mutan	2.12
87	63	51	Senior high school	Housewife	161	70.1	27.1	obesity	2	(-)	591.84	68.23	AA	Wild Type	1.71
88	64	41	elementary school	Housewife	155	70.7	29.5	obesity	1	(+)	352.368	58.601	GG	Mutan	2.51
89	66	64	Junior high school	Housewife	156	71.3	29.3	obesity	2	(-)	505.674	99.95	AG	Mutan	5.81
90	67	70	elementary school	Housewife	150	70	31.1	obesity	2	(-)	172.998	47.465	GG	Mutan	15.24
