# Supplementary material for: Risk factor of elevated matrix metalloproteinase-3 gene expression in synovial fluid in knee osteoarthritis women
Source: PLoS One. 2023 Mar 31;18(3):e0283831. doi: 10.1371/journal.pone.0283831 (PMC10065264; doi:10.1371/journal.pone.0283831)
Supplement: Attachment — Submitted filename: Response to Reviewer.docx [file pone.0283831.s002.docx]

The Rebuttal Letter

Dear author and reviewer,

This is my rebuttal letter in respond to your advice.

1. I have ensured my manuscript meets PLOS ONE’style requirement.
2. I corrected a few words or sentences that had grammatical or tense errors; you can check them in the revised manuscript with track change
3. This research is a continuation of previous research that has been published in https://oamjms.eu/index.php/mjms/article/view/8985

I have added on the reference list

4. I added and ensured about informed consent sheet in methode, page 7 (revised manuscript with track change)
5. I’ve included the amended Role of Funder statement in my cover letter about financial disclosure. "The funders had no role in study design, data collection and analysis, decision to publish, or preparation of the manuscript."
6. I have fixed tahe acknowledgments section in page 24 (revised manuscript with track change)
7. Response to reviewer’s comment:

| No. | Reviewer’s comments | Response | Location |
| --- | --- | --- | --- |
| 1. | Title and elsewhere throughout MS, as well) Please, do not use acronyms in headings | I have corrected the title. I've removed the acronym in the heading. | Title |
| 2. | (References) 1. … N. Classifications in Brief: Kellgren-Lawrence Classification of Osteoarthritis… | From this reference source, I took the definition of osteoarthritis in the article. So, I don't think it needs to be fixed. | Pag1e 1(revised manuscript with track change) |
|  | (Introduction, 7th paragraph and elsewhere throughout MS, as well) please, do not start sentences with acronyms | I've removed the acronym from the entire beginning of the sentence. | Page 5 (revised manuscript with track change) |
|  | Introduction (10th par) please, check for font size; | I’ve checked and fixed the font size | Page 6 (revised manuscript with track change) |
|  | (Materials and Methods, 3rd par) The CT method… | I’ve checked and fixed that. | Page. 9 (revised manuscript with track change) |
|  | (Age with MMP 3 Gene Expression rs679620, 1st-2nd par) please, check for correct in-text referencing; ref Komosinska-Vassev et al. not in References; | I have fixed and added the new reference. | Page 14 (revised manuscript with track change) |
|  | (3rd par) is aggrecan an advanced glycation end product? Please, explain; | I've added an explanation of this, as well as added a new reference | Page 15 (revised manuscript with track change) |
|  | (Polymorphism with MMP 3 Gene Expression rs679620, 1st par) please, introduce CEU; | I’ve added the meaning of CEU. It stands for central Europe. | Page 16 (revised manuscript with track change) |
|  | (2nd÷5th par) please, check for correct in-text referencing; Almeida et al. (2014)≠(30); | I’ve fixed that. | Page 17 (revised manuscript with track change) |
|  | (Levels of IL-1β and TNF-α with MMP3 Gene Expression rs679620, 1st-2nd par) please, check for correct in-text referencing; … in OA. Under… | I think the reference source is correct | Page 18 (revised manuscript with track change) |
|  | (Menopause Status with MMP 3 Gene Expression rs679620, 2nd par) please, check for correct in-text referencing. | I’ve fixed that | Page 20 (revised manuscript with track change) |
